# Mechanical Intermittent Compression Affects the Progression Rate of Malignant Melanoma Cells in a Cycle Period-Dependent Manner

**DOI:** 10.3390/diagnostics11061112

**Published:** 2021-06-18

**Authors:** Takashi Morikura, Shogo Miyata

**Affiliations:** 1 Graduate School of Science and Technology, Keio University, Yokohama 223-8522, Japan; dnngu-1elife@keio.jp; 2 Department of Mechanical Engineering, Faculty of Science and Technology, Keio University, Yokohama 223-8522, Japan

**Keywords:** mechanical intermittent compression, malignant melanoma, in vitro model, cancer progression

## Abstract

Static mechanical compression is a biomechanical factor that affects the progression of melanoma cells. However, little is known about how dynamic mechanical compression affects the progression of melanoma cells. In the present study, we show that mechanical intermittent compression affects the progression rate of malignant melanoma cells in a cycle period-dependent manner. Our results suggest that intermittent compression with a cycle of 2 h on/2 h off could suppress the progression rate of melanoma cells by suppressing the elongation of F-actin filaments and mRNA expression levels related to collagen degradation. In contrast, intermittent compression with a cycle of 4 h on/4 h off could promote the progression rate of melanoma cells by promoting cell proliferation and mRNA expression levels related to collagen degradation. Mechanical intermittent compression could therefore affect the progression rate of malignant melanoma cells in a cycle period-dependent manner. Our results contribute to a deeper understanding of the physiological responses of melanoma cells to dynamic mechanical compression.

## 1. Introduction

Malignant melanoma is a melanocyte-derived cutaneous skin tumor, which is known as one of the most aggressive cancers and intractable disease with a poor prognosis [1]. The incidence of malignant melanoma is increasing worldwide [2,3,4], but there are few effective pathological diagnostic techniques to find melanoma [5]. Although a classical “ABCDE” approach is generally considered useful for pathological evaluation of major melanoma subtype such as superficial spreading melanoma (SSM), the pathological evaluation of specific minor subtype is difficult, such as acral lentiginous melanoma (ALM) because the lesions are often heterogeneous and unique [6,7,8]. The difficulty in diagnosing delays the early detection of lesions, and the associated mortality rate is high because the stage of the disease is often advanced at the time of detection [9,10].

In addition to the lack of effective diagnostics, establishing an effective therapeutic strategy without adverse events remains challenging [11]. Most of the currently available clinical therapies have been developed for major melanoma subtypes, such as SSM, which often occurs in the UV-exposed skin, and there are very few effective treatments for minor subtypes such as ALM, which often occurs on the plantar surface [12,13]. For instance, the molecular targeted drugs and immune checkpoint inhibitors that target mutation, such as BRAF and NRAS gene, are effective for SSM, but ALM has a poor response to these treatments because there are relatively few above-mentioned genetic mutations [14,15,16,17]. Therefore, the development of effective treatment and diagnostic strategy for minor melanoma subtypes, such as ALM, is also vitally important.

To establish effective new therapies and diagnostic techniques for cancer, it is important to elucidate the progression mechanisms of the targeted cancer. The development and progression of superficial spreading melanoma, which is common in Caucasoid patients, is correlated with UV exposure, which can cause genetic mutations, such as BRAF and NRAS mutations, which lead to the development and progression of malignant melanoma [18,19,20]. However, UV exposure of the plantar surface, where ALM commonly occurs, is limited, and some studies have suggested that there is a different mechanism in the progression of melanoma than the genetic mutations caused by UV exposure [12,21]. Recently, the physical environment surrounding malignant melanomas and mechanical stimulus have attracted attention, and the relationship between mechanical stimulation and the progression of malignant melanoma cells has been highlighted.

During tumor growth, cancer cells invade the surrounding interstitial tissue and distantly metastasizes to other tissues by passing through the extracellular matrix (ECM) to infiltrate blood vessels or lymphatics [22,23,24]. During interstitial tissue invasion, cancer cells are exposed to a variety of mechanical stimuli, such as compression, tension, and shear stimuli. Interestingly, some studies have reported that the behavior of cancer cells changed to adapt to external mechanical stimuli as a biochemical response. Cheng et al. reported that microenvironmental mechanical stimuli regulate tumor size and morphology by inhibiting cell proliferation and promoting apoptosis [25], while Janet et al. showed that mechanical compression contributes to the acquisition of invasive capabilities by cancer cells [26]. Similarly, several previous studies on malignant melanoma have also reported a relationship between the mechanical environment and cancer progression. Importantly, there seems to be a correlation between the area of the plantar surface, where strong mechanical stimuli are applied, and the site of malignant melanoma development, with malignant melanoma size being more expanded in areas under more intense mechanical stimuli [27,28]. We previously reported that static mechanical compression promotes melanoma cell invasion [29]. Those reports suggest that mechanical compression lead a biochemical response associated with progression of melanoma cells. However, little is known about how dynamic mechanical compression affects the progression of melanoma cells. Therefore, in the present study, we investigated the effect of mechanical intermittent compression on the progression of melanoma cells as fundamental research.

The aim of the present study was to elucidate how mechanical intermittent compression affects the progression of malignant melanoma cells in a cell culture model simulating physiological conditions. We established an in vitro cell culture model and cell culture device to apply the mechanical intermittent compression with temporal observation. After the establishment of the cell culture system, the effect of mechanical intermittent compression on the progression of melanoma cells was evaluated.

## 2. Materials and Methods

### 2.1. In Vitro Malignant Melanoma Model to Enable Mechanical Intermittent Compression and Temporal Observation of Cell Behavior

A mouse malignant melanoma cell line (B16F10, RIKEN BioResource Center, Tsukuba, Japan) was used to establish an in vitro malignant melanoma model. B16F10 cells were thawed from cryopreserved stock and subcultured twice in Dulbecco’s modified Eagle’s medium (DMEM)-high glucose, supplemented with 10% fetal bovine serum and 1% antibiotics/antimycotics. The cells were maintained in a 5% CO_2_ atmosphere at 37 °C and passaged once in 2–3 days to avoid reaching confluence, which inhibited cell-cell contact.

**Figure 1:**
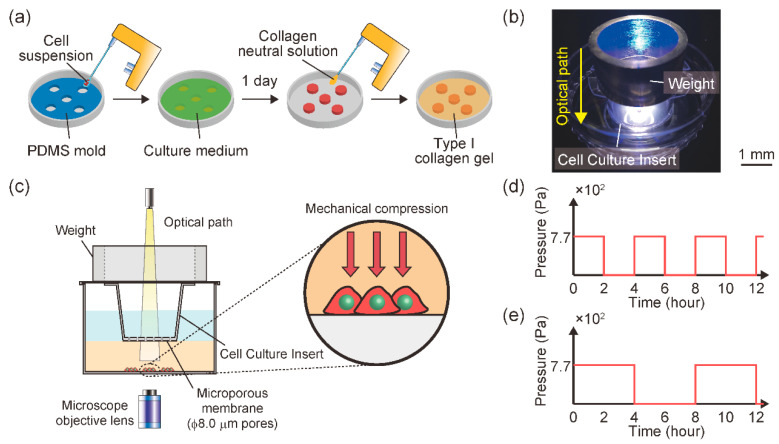
Schematic of the in vitro malignant melanoma model. (**a**) Fabrication of the in vitro malignant melanoma model. B16F10 cells were seeded in a 1.5 mm cylindrical area in the PDMS mold on a cell culture dish. After one day of culture, the mold was removed from the dish and covered with neutralized type I collagen gel. (**b**) Photograph of the experimental set-up. (**c**) Schematic side view of experimental set-up for imposing intermittent mechanical compression and monitoring the cell behavior. (**d**) Mechanical intermittent compression pattern of T = 4 groups. (**e**) Mechanical intermittent compression pattern of T = 8 groups.

An in vitro malignant melanoma model was established in our previous study [29]. Briefly, the model was established by seeding B16F10 cells under a type I collagen gel layer, simulating dermal tissue. B16F10 cells were seeded at 1.6 × 10^5^ cells/cm^2^ in a 1.5 mm cylindrical area on an f 60 mm cell culture dish (Figure 1a). A type I collagen neutral solution was prepared at a final concentration of 2.4 mg/mL from acid-soluble collagen (I-AC30, KOKEN, Tokyo, Japan). Type I collagen solution (2 mL) was poured into f 60 mm cell culture dishes to cover the B16F10 cells. After polymerization at 37 °C for 20 min, a Cell Culture Insert (pore size; f 8.0 mm, BD Falcon Inc, Franklin Lakes, NJ, USA) was mounted on the gel layer to permit oxygen and nutrient diffusion toward the B16F10 cell-seeded area. The malignant melanoma model was maintained in DMEM-high glucose with 10% FBS and 1% antibiotics/antimycotics at 37 °C in 5% CO_2_ for 72 h.

A cell culture device was also established to enable intermittent mechanical compression with temporal observation (Figure 1b). To impose mechanical compression onto the gel-covered cells, a cell culture insert with a cylindrical SUS304 weight was mounted on the gel layer (Figure 1c). B16F10 cells were compressed through the collagen gel layer using the Cell Culture Insert with a ring-shaped weight. The melanoma model was subjected to a mechanical intermittent compression of 7.7 × 10^2^ Pa with a cycle of 2 h on/2 h off (T = 4 groups) (Figure 1d) or 4 h on/4 h off (T = 8 groups) (Figure 1e).

Gene expression related to cellular behavior, such as invasion and cell proliferation, fluctuates over time. Gene expression in response to sustained mechanical stimulation is transient, and stabilizes within a few hours. Therefore, we prepared two sample groups that switched mechanical stimuli at the level of several hours. A malignant melanoma model without weights was also prepared similarly for use as the control.

### 2.2. Creep Phenomenon of Collagen Gel in the Cell Culture Device during Application of Continuous Mechanical Compression

For evaluating the creep in our experimental system, a type I collagen gel containing f 20 mm polystyrene microspheres (Polybead; 18329, Polysciences Inc., Warrington, PA, USA) was prepared. Briefly, a type I collagen neutral solution was prepared at a final concentration of 2.4 mg/mL from I-AC30 acid-soluble collagen. The collagen-neutral solution was mixed with 20 mm polystyrene microspheres to yield a final concentration of 5 *v*/*v*% of microspheres. The microspheres were used as markers to evaluate the creep phenomenon of the collagen gel under compression. The type I collagen solution (2 mL) was poured into f 60 mm cell culture dishes, and polymerized at 37 °C for 20 min, and the cell culture insert and ring-shaped weight were mounted on the gel layer, similar to the cell culture experiments.

**Figure 2:**
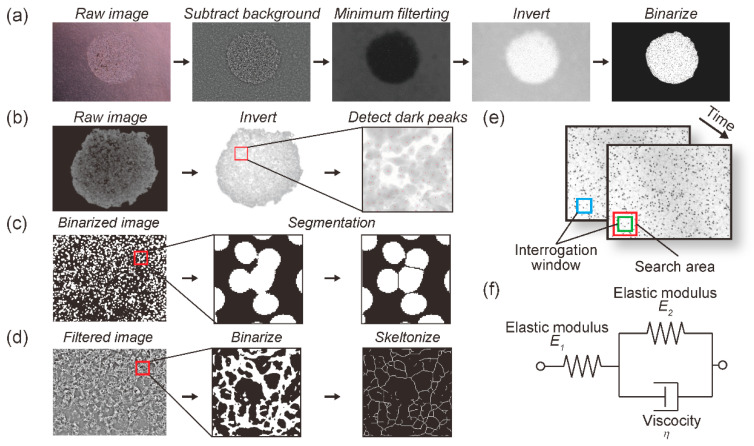
Image analysis and evaluation of creep phenomenon: (**a**) Quantification of cell-occupied area to evaluate cell progression. (**b**) Enumeration of live cells using the ITCN plugin in ImageJ. (**c**) Enumeration of nuclei using binarization and segmentation (**d**) Quantification of total F-actin length using binarization and skeletonization. (**e**) Schematic of digital image correlation method (DIC) (**f**) Three-element generalized Kelvin-Voigt model.

The prepared creep test specimens were subjected to compressive stimulation for 30 min. Time-lapse images were acquired every minute using a phase contrast microscope (CKX41, Olympus Inc., Tokyo, Japan) equipped with a CCD camera (DP73, Olympus Inc., Tokyo, Japan). Using these images, the temporal strain change in the collagen gel was measured using digital image correlation (DIC), which is a contact-free measurement of material deformation [30,31]. The strain in the collagen gel was measured according to the DIC algorithm at each time point before and after deformation as follows: (1) an interrogation window was set in an arbitrary search area at each time point (Figure 2e). (2) The cross-correlation coefficients of the pixel value pattern in the interrogation window before and after deformation were calculated. (3) The location of the interrogation window where the cross-correlation was maximum was measured as the location after deformation. (4) The displacement of the location between the set interrogation window before and after deformation was calculated as the deformation. (5) Using the measured deformation magnitude, the Green-Lagrange strain was calculated, which contains normal strain and shear strain variables. The normal strain and shear strain in the collagen gel were measured according to the DIC algorithm at each time point before and after deformation, and given as the Green-Lagrange strain. The temporal strain change was measured using the open-source software package Ncorr [32] in the numerical analysis software MATLAB (9.9.0.1570001 (R2020b), MathWorks, Natick, MA, USA).

After the representative strain value, defined as the squared norm of the median value of the normal strain in the analyzed area, was calculated, a creep curve was generated. The creep phenomenon of biomaterials, such as biological tissue and collagen gel, is generally described using the generalized Kelvin-Voigt model [33,34]. Nonlinear regression of the creep curve of each sample was performed using a three-element model (Figure 2f). The three-element generalized Kelvin-Voigt model is described as follows:

(1) $$\tau =\frac{\eta }{E_{2}}$$

(2) $$\gamma =\frac{\sigma_{0}}{E_{1}}+\frac{\sigma_{0}}{E_{2}}\left(1-e^{-\frac{t}{\tau }}\right)$$

where γ is the strain, $\sigma_{0}$ is the applied constant stress, $E_{i}\;\left(i=1,2\right)$ is the elastic modulus for each component, and η is the viscosity. The delay time τ of the model, which is defined as Equation (1), was estimated using the Levenberg-Marquardt method in the open-source statistical analysis software R. The fit index between the creep curve and the estimated nonlinear curve using the three-element model was evaluated using Pearson’s correlation coefficient.

### 2.3. Quantification of Cell Progression

The cell behavior was observed for 24 h using a phase-contrast microscope (CKX41, Olympus Inc., Tokyo, Japan) equipped with a CCD camera (DP73, Olympus Inc., Tokyo, Japan). Phase contrast images were continuously acquired at 0 h, 8 h, 16 h, and 24 h under mechanical intermittent compression. Progression was evaluated using the progression distance (l) in the phase-contrast images, which was measured using ImageJ software. To remove the noise within the phase-contrast images, pre-processing was conducted, including filtering and binarization (Figure 2a). The progression distance at each time point $\left(l_{t}\right)$ was calculated as follows:

(3) $$l_{t}=\sqrt{\frac{a_{t}}{\pi }}-\sqrt{\frac{a_{0}}{\pi }}\;$$

where $a_{t}$ is the cell-occupied area at each time and $a_{0}$ is the area at 0 h. The radius of the approximate perfect circle, which is equivalent to the cell-occupied area, was calculated, and the difference between the radius of the perfect circle approximating the cell-occupied area at each time and that at the start of culture was defined as the cell progression distance $\left(l_{t}\right)$.

### 2.4. Cell Viability and Cell Proliferation Assay

To determine the effect of mechanical intermittent compression on cell viability and cell proliferation rate in the malignant melanoma model, a fluorescence live/dead assay was performed after 24 h of culture. The cells were characterized using calcein AM/propidium iodide (PI) double fluorescence staining.

Cell viability was defined as the dead cell rate (DCR), which was calculated as follows:

(4) $$DCR=\frac{N_{L}}{N_{L}+N_{D}}\;$$

where $N_{L}$ is the number of live cells and $N_{D}$ is the number of dead cells at the end of the culture duration. The number of viable cells ($N_{L}$) was measured using the ITCN plugin in ImageJ (Figure 2b). The number of dead cells ($N_{D}$) was measured using ImageJ according to the following: (1) grayscale images were binarized using the *Otsu* algorithm, and (2) the nucleus area was segmented using the *watershed* algorithm (Figure 2c).

The cell proliferation rate (CPR) was calculated as follows:

(5) $$CPR=\frac{N_{L}+N_{D}}{{\left(N_{L}+N_{D}\right)}_{control}}\;$$

where $N_{L}$ and $N_{D}$ were calculated using the same measurement method as the cell viability assay, and ${\left(N_{L}+N_{D}\right)}_{control}$ was defined as the sum of $N_{L}$ and $N_{D}$ in the control group.

### 2.5. Fluorescence Staining of F-Actin and Nuclei

To determine the effect of mechanical intermittent compression on the morphological changes in F-actin filaments in the cell-occupied area, the morphology of F-actin filaments was observed by rhodamine-phalloidin/DAPI fluorescence double staining at 24 h of culture. Briefly, cells in the malignant melanoma model were fixed with 4% paraformaldehyde for 10 min. The cells were then permeabilized with 0.1% Triton X-100 in PBS for 5 min. To stain F-actin filaments, the cells were incubated with 0.7% rhodamine-phalloidin (PHDR1, Cytoskeleton Inc., Denver, CO, USA) for 30 min at 37 °C. After rhodamine-phalloidin staining, 300 nM DAPI solution was added and incubated for 5 min. After removing the DAPI solution, the cells were rinsed with PBS + 1% antimycotic/antibiotic for 5 min three times. F-actin and DAPI were visualized using an inverted fluorescent microscope (CKX41, Olympus Inc., Tokyo, Japan) equipped with a CCD camera (DP73, Olympus Inc., Tokyo, Japan) and fluorescent equipment (U-LH50HG, Olympus Inc., Tokyo, Japan). The length of F-actin filaments was measured to quantitatively evaluate morphological changes in the cytoskeleton. The length of F-actin filaments per single cell (LFC) was calculated as follows:

(6) $$LFC=\frac{L_{f}}{N_{n}}\;$$

where $L_{f}$ and $N_{n}$ are the total actin fiber length and number of cell nuclei per acquired image at the end of the culture duration, respectively. The number of cell nuclei per acquired image ($N_{n}$) was measured using ImageJ according to the following: (1) grayscale images were binarized using the *mean* algorithm, and (2) the nucleus area was segmented using the *watershed* algorithm (Figure 2c). The total actin fiber length per acquired image ($L_{f}$) was measured using ImageJ according to the following: (1) acquired images were pre-processed by a bandpass filter for noise removal and edge-enhancement, (2) grayscale images were binarized using the *Otsu* algorithm, (3) pixels were repeatedly removed from the edges of objects in the binary image until they were reduced to single-pixel-wide shapes, (4) the sum of grayscale in the skeletonized images was equivalent to the total actin fiber length per acquired image (Figure 2d).

### 2.6. Relative Quantification of mRNA Expression Levels

The relative mRNA expression levels in the cell culture model were quantified by RT-qPCR for matrix metalloproteinase-14 (*Mmp-14*) and glyceraldehyde 3-phosphate dehydrogenase (*Gapdh*), encoding MMP14 and GAPDH, respectively. *MMP14* is a key ECM-degrading enzyme, and also a regulator that activates proteins that promote the progression of melanoma cells [35]. GAPDH is a crucial factor in glycolysis and is one of the most commonly used reference genes [36].

Relative mRNA expression levels were measured using total RNA extracted from the cell culture model collected after 24 h of culture. Total RNA was extracted using NucleoSpin RNA kits (740955.50; Takara Bio Inc., Shiga, Japan) and quantified using a Thermal Cycler Dice Real Time System Lite (TP700; Takara Bio Inc., Shiga, Japan). RNA was reverse transcribed into cDNA using the PrimeScript Master Mix (Perfect Real Time) (RR036A; Takara Bio Inc., Shiga, Japan) with an oligo (dT) primer and random hexamer primer for 15 min at 37 °C and 5 s at 85 °C. The concentration of cDNA was quantified using a Biophotometer (6131; Eppendorf, Hamburg, Germany), and then diluted with RNase-free water (9012; Takara Bio Inc., Shiga, Japan) to 10 ng/mL of cDNA. RT-qPCR was conducted in a Thermal Cycler Dice Real Time System Lite using the PCR program 30 s at 95 °C, followed by 60 cycles of 5 s at 95 °C and 30 s at 60 °C. The RT-qPCR reaction mix contained 12.5 µL of TB Green Premix Ex Taq II (Tli RNaseH Plus) (RR820A; Takara Bio Inc., Shiga, Japan), 20 ng of cDNA, 0.4 mM of each forward and reverse primer, and 8.5 µl of RNase-free water. The primer sequences are listed in Table 1. RT-qPCR was performed in technical triplicates for each primer pair and cDNA sample. In addition, the reactions were conducted in biological triplicates under similar conditions. To verify that primer dimers were not responsible for the obtained fluorescence signals, melting curve analysis of the amplicons was performed for each primer pair. Negative control reactions without templates were also included to ensure data quality. Relative mRNA expression was normalized to GAPDH and then calibrated to that of the control group. The fold change was calculated using the $2^{-\Delta \Delta C_{t}}$ method, where $C_{t}$ is the threshold cycle.

### 2.7. Statistical Analysis

The statistical significance of the differences between experimental groups was evaluated using Dunnett’s test. Statistical significance was set at *p* < 0.05 and *p* < 0.001.

## 3. Results

### 3.1. Establishment of a Cell Culture Device to Apply Mechanical Intermittent Compression with Temporal Observation

We established a cell culture device to apply mechanical intermittent compression with temporal observation. Prior to observing the progression of melanoma cells in the cell culture device, we evaluated the deformation of collagen gel during applying mechanical compression; the creep phenomenon of the gel.

**Figure 3:**
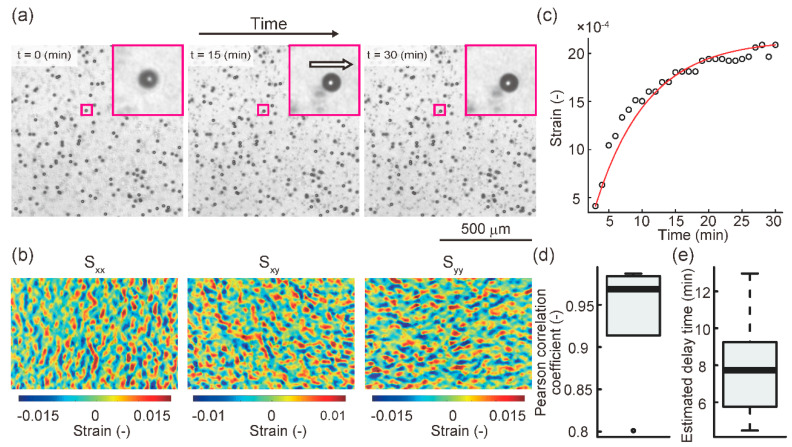
Creep estimation in the collagen gel during mechanical compression: (**a**) Representative time-lapse images acquired by phase contrast microscopy. The black dot object in the image indicates a polystyrene microsphere. The white arrow indicates the direction of displacement. (**b**) Representative images of Green-Lagrange strain. $S_{xx}$ indicates a horizontal normal strain, $S_{yy}$ indicates a vertical normal strain, and $S_{xy}$ indicates a shear strain. (**c**) Representative creep curve and estimated nonlinear curve fitted by the three-element Kelvin-Voigt model. (**d**) Boxplot of Pearson’s correlation coefficients. The median of the Pearson’s correlation coefficients between the creep curves and the estimated nonlinear curves was 0.968 (n = 6). (**e**) Boxplot of estimated delay time. The median estimated delay time was 7.74 min (n = 6).

Representative images of the deformation and strain distribution in the collagen gel are shown in Figure 3a,b. Figure 3c shows the creep curve during mechanical compression and an estimated nonlinear curve fitted by the three-element Kelvin-Voigt model. Regarding the fit index of nonlinear regression, the median of the Pearson correlation coefficients between the creep curves and the estimated nonlinear curves using the generalized Kelvin-Voigt model was 0.968 (Figure 3d). This indicates that the estimation of the creep curve using the models fitted well. As a result of nonlinear regression, the median of the estimated delay time was 7.74 min, which is very small compared to the application time of compression, which was 120 min (Figure 3e). Based on these results, the creep phenomenon horizontal to the surface of the cell culture dish during temporal compression was negligible.

### 3.2. Progression Rate of Cells in Melanoma Model Was Regulated by Mechanical Intermittent Compression in a Cycle Period-Dependent Manner

**Figure 4:**
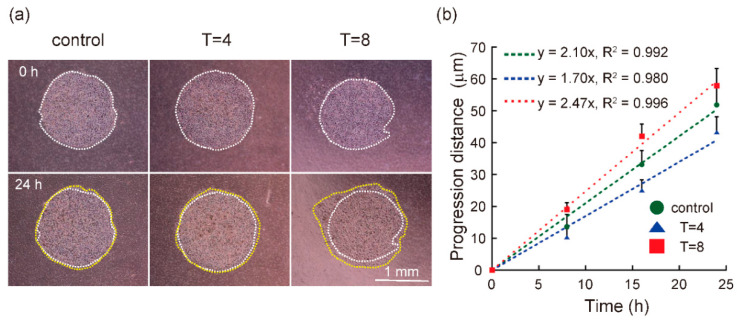
Progression of B16F10 cells in a malignant melanoma model under mechanical intermittent compression. (**a**) Representative phase contrast image. The white dotted lines indicate the cell-adhered area at 0 h of culture, and the yellow dotted lines indicate the cell-adhered area at 24 h of culture. (**b**) Quantification of progression distance. The green circles indicate the progression distance in the control group, the blue triangles indicate the T = 4 group, and red rectangles indicate the T = 8 group. The green, blue, and red dashed lines indicate a regression line to the progression distance in the control, T = 4, and T = 8 groups, respectively (n ≥ 12, data represents the mean ± S.E).

Representative microscopic images of B16F10 cells in the control, T = 4, and T = 8 groups are shown in Figure 4a. The white dotted line indicates the cell-occupied area at 0 h of culture, and the yellow dotted line indicates the cell-occupied area at 24 h of culture. The cell-occupied area in the established cell culture model increased during the cultivation period. Figure 4b shows the progression distance at each time, $\left(l_{t}\right)$, during the cultivation period. The slope of the progression distance in the T = 4 group was lower than that in the control group. In contrast, the slope in the T = 8 group was higher than that in the control group. In other words, the progression rate in the T = 4 group decreased, whereas that in the T = 8 group increased. This suggests that intermittent compression with a cycle of 2 h on/2 h off could suppress the progression rate of melanoma cells, while a cycle of 4 h on/4 h off could promote the progression rate.

### 3.3. Cell Viability and Cell Proliferation Rate

**Figure 5:**
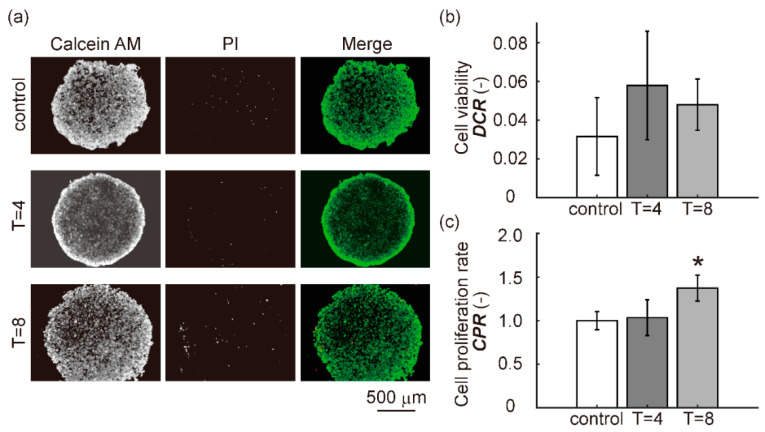
Cell viability and cell proliferation assay. (**a**) Representative fluorescent images stained by calcein AM/PI at 24 h culture duration. The green fluorescence indicates live cells, and the red fluorescence indicates dead cells. (**b**) Quantification of cell viability (*DCR)* (n = 3, mean ± S.D.). (**c**) Quantification of cell proliferation rate (*CPR)* (n = 3, mean ± S.D.). Dunnett’s test was used to compare groups. * indicates a significant difference compared to the control group (*p* < 0.05).

Representative fluorescence double staining images using calcein-AM/PI in the control, T = 4, and T = 8 groups are shown in Figure 5a. Most cells in all groups were alive after 24 h of culture. Figure 5b shows the quantitative cell viability, defined as the dead cell rate (DCR). Figure 5c shows the quantitative cell proliferation rate (CPR). There was no significant difference in DCR between the control groups and the T = 4 and T = 8 groups. There was no significant difference in CPR between the control and T = 4 groups, while the CPR in the T = 8 group increased significantly compared to that in the control group. These findings suggest that intermittent compression with a cycle of 2 h on/2 h off did not affect cell viability and proliferation. In contrast, intermittent compression with a cycle of 4 h on/4 h off did not affect cell viability, but promoted cell proliferation.

### 3.4. Cell Migration Capacity

**Figure 6:**
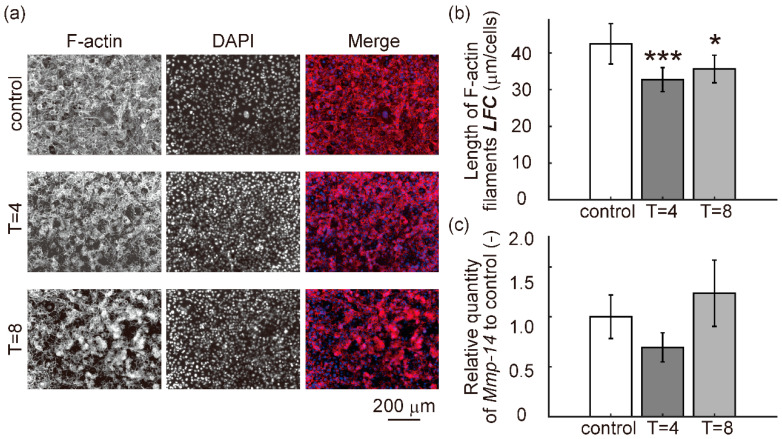
Quantification of cell migration and invasion capacity. (**a**) Representative fluorescence images stained by rhodamine-phalloidin/DAPI at 24 h of culture. The red fluorescence indicates F-actin filaments, and the blue fluorescence indicates nuclei. (**b**) Quantification of the length of F-actin filaments (*LFC*) (n ≥ 3, mean ± S.D.). (**c**) Relative quantity of *Mmp-14* (n = 3, mean ± S.D.). Dunnett’s test was used to compare groups. Asterisks indicate a significant difference compared to the control group (*: *p* < 0.05, ***: *p* < 0.001).

Representative rhodamine-phalloidin/DAPI fluorescence staining images in the control, T = 4, and T = 8 groups are shown in Figure 6a. Figure 6b shows the value of the *LFC*, which was defined as the length of F-actin filaments. The *LFC* in the T = 4 group decreased significantly compared to that in the control group, and there was significant decrease between the *LFC* values in the control and T = 8 groups. The LFC in the T = 4 group tended to decrease compared to that in the T = 8 group. In general, elongation of F-actin filaments is correlated with cell motility [37,38,39]. These results suggest that intermittent compression with a cycle of 2 h on/2 h off could suppress the cell migration capacity rather than a cycle of 4 h on/4 h off.

### 3.5. Relative mRNA Expression Levels

The relative mRNA expression levels of *Mmp-14* are shown in Figure 6c. The mRNA expression of *Mmp-14* in the T = 4 group was lower than that in the control group, while that in the T = 8 group increased compared to in the control group. Mechanical intermittent compression with a cycle of 2 h on/2 h off might suppress the invasion ability of melanoma cells by regulating the expression of *Mmp-14*. In contrast, compression with a cycle of 4 h on/4 h off might activate the invasive ability of melanoma cells.

## 4. Discussion

Pathological diagnosis of minor melanoma subtype, which often occurs on the soles of feet, is difficult compared to other melanoma types [6,7,8]. In addition, the minor subtype responds poorly to current therapy strategies [14,15,16]. For these reasons, it is important to elucidate the mechanisms by which these minor melanoma progress to establish new pathological diagnostic strategies and therapies. Interestingly, although ultraviolet light is generally thought to be a factor in the development of melanoma, mechanical stimuli may affect the development and progression of melanoma as well as genetic damage caused by UV exposure [12,21]. We previously reported that static mechanical compression promotes the progression of melanoma cells [29]. However, little is known about how dynamic mechanical compression, such as intermittent compression, affects the progression of melanoma cells. The aim of the present study was therefore to elucidate the mechanisms by which mechanical intermittent compression affects the progression of melanoma cells.

We established a cell culture model simulating the physiological conditions of melanomas, and a cell culture device to apply intermittent mechanical compression with temporal observation. In general, it is known that creep occurs when a continuous mechanical force is applied to a viscoelastic material. As a result of the creep phenomenon, the material deforms gradually under continuous force. In the established cell culture device, creep deformation occurred horizontal to the surface of the cell culture dish when a compressive stimulus was applied. The horizontal deformation in response to mechanical compression could apply shear stress to the melanoma cells. If the shear stress is not negligible, it may be a confounding factor in the elucidation of the effects of mechanical intermittent compression on the progression of melanoma cells. Therefore, we evaluated the creep phenomenon of collagen gel in cell culture devices under compression. When the delay time of the creep phenomenon was negligible compared to the observation time, we assumed that the shear stress that the cells were subjected to was also negligible. We measured creep in the collagen gel, and showed that the creep phenomenon in the horizontal direction of the culture dish that was caused by continuous applied compression was negligible. Based on this, we can assume that the effect of intermittent compressive stimulation on melanoma cells can be measured because shear stimulation caused by gel creep can be ignored in the established cell culture device.

We showed that mechanical intermittent compression affects the progression rate of melanoma cells in a cycle period-dependent manner. Interestingly, we found that intermittent compression with a cycle of 2 h on/2 h off suppressed the progression rate of melanoma cells. Under these conditions, the length of F-actin filaments decreased and the mRNA expression level of *Mmp-14*, which is related to collagen degradation, decreased. In general, the cytoskeleton, including F-actin filaments, is reorganized and elongated in the direction of cell migration [40,41,42,43]. In other words, the suppression of F-actin filament length correlates with decreased cell motility. In addition, the gene expression level of *Mmp-14*, which promotes collagen degradation, correlates with the invasive ability of melanoma cells in collagen gel [35]. Taken together, these findings suggest that intermittent compression with a cycle of 2 h on/2 h off reduced the progression rate by decreasing the cell migration capacity and invasive ability of melanoma cells through the inhibition of F-actin elongation and collagen degradation, respectively. Here, we should note that the morphological analysis algorithm developed for F-actin has some advantages and limitations compared to conventional analysis approaches. In general, Evaluation of the single-cell level is required to quantify the change of the cytoskeletal morphology. However, in cell culture model simulating biological tissue with high cell density such as our model, it is extremely difficult to segment them even with the advanced mathematical models and machine learning techniques because the cells overlap each other [44]. Hence, to evaluate the morphological changes of the cytoskeleton in the high cell density area, such as our established model, we developed an algorithm to extract the bulk morphological features of the cytoskeleton at the multi-cell level. It has the advantage of being able to measure changes in the cytoskeleton even in regions of high cell density, and the analysis results using our algorithm are sufficient to evaluate the effects between the different stimulus conditions as a fundamental study. On the other hand, the algorithm does not allow for a detailed evaluation of various actin morphologies, such as the filamentous and globular actin. To gain a deeper understanding of cytoskeletal responses under the dynamic mechanical compression, immunofluorescence staining and protein expression analysis are necessary, which is our future work.

In contrast, intermittent compression with a cycle of 4 h on/4 h off could promote the progression rate of melanoma cells. The increasing of the cell-occupied area in the established cell culture model, which indicates the progression of melanoma cells, is caused by the synergistic interaction between cell proliferation, migration, and invasion. The combined effect of those factors causes an increase in the cell-occupied area. As shown in the results from the molecular biological evaluation and image analysis, the cell proliferation and mRNA expression level of *Mmp-14* increased in T = 8 groups. It is also known that the increased mRNA expression level of *Mmp-14* correlates with the promotion of invasion via collagen degradation [45]. Some studies have reported that the collagen degradation by *Mmp-14* is crucial for cancer cells to proliferate and invade in the ECM [46,47,48]. Shaverdashvili et al. showed that *Mmp-14* is directly contributed to the metastasis of melanoma [49]. In addition, although *Mmp-2* and *Mmp-9* are known to play important roles in the migration and invasion processes of melanoma, *Mmp-14* can activate both [50,51]. Thus, *Mmp-14* is a critical factor in the progression process of melanoma. The result that the expression of *Mmp-14* increased in our melanoma model suggests promoting the progression of melanoma cells. To elucidate the physiological mechanisms of the melanoma progression under the conditions in more detail, the gene expression analysis of other mRNA and protein, such as gene related to cytoskeleton reconstruction, and the metabolic measurements such as glucose consumption are required. In summary, intermittent compression with a cycle of 4 h on/4 h off might promote the progression rate of melanoma cells by accelerating the increase in cell number and invasive ability through the promotion of cell proliferation and collagen degradation, respectively.

We showed for the first time that mechanical intermittent compression affects melanoma cell invasion in a cycle period-dependent manner in this study. However, we should notice that the cell line used in this study is a mouse melanoma cell line, not a human cell line. It is necessary to determine whether the mechanical intermittent compression can affect human melanoma similarly in future work, such as human melanoma cell line and primary melanoma cells collected from the patients. Also, to understand deeply the molecular biological mechanisms more, it is required to conduct comprehensive gene expression analysis and metabolic measurement, and cell culture experiments under cyclic compressive stimulation with different time resolutions in the future.

It may be possible to regulate the invasion of melanoma cells by applying mechanical compressive stimuli with appropriate cycle periods. Mechanical stimuli can be controlled less invasively and more precisely than pharmacokinetic or electromagnetic field control methods. Thus, our results may contribute to establish new therapies that are less invasive and more locally effective than conventional therapies, such as drug therapy, surgery, and radiotherapy. In addition, if a unique relationship between the mechanical stimulation pattern and the progression rate of melanoma is found, new criteria for pathological diagnostic techniques could be established. Our results have the potential to contribute to the establishment of new diagnostic and therapeutic methods.

## 5. Conclusions

We established an in vitro cell culture model using melanoma cells to simulate the physiological conditions of malignant melanoma, and a cell culture device to apply intermittent mechanical compression with temporal observation.

In the present study, mechanical intermittent compression with a cycle of 2 h on/2 h off could suppress the progression rate of melanoma cells, by suppressing the elongation of F-actin filaments and regulating the levels of mRNA related to collagen degradation. In contrast, mechanical intermittent compression with a cycle of 4 h on/4 h off could promote the progression rate of melanoma cells by promoting cell proliferation and regulating the levels of mRNA related to collagen degradation.

In conclusion, our study revealed that the mechanical intermittent compression affected the progression of melanoma cells in a cycle period-dependent manner. The result will lead to a deeper understanding of melanoma cell behavior under dynamic mechanical compression and could contribute to the establishment of new diagnostics and therapy.

## Figures and Tables

**Table 1:** RT-qPCR primer sequences.

Gene Name	Gene Bank Accession Number	Sequence(5′-3′)	Tm (°C)	Product Size (bp)
***Gapdh***	NM_001289726.1	Forward TGTGTCCGTCGTGGATCTGA Reverse TTGCTGTTGAAGTCGCAGGAG	63.9 63.9	3939
***Mmp-14***	NM_008608.4	Forward CCTCAAGTGGCAGCATAATGAGA Reverse TGGCCTCGAATGTGGCATAC	63.7 64.3	83

## References

[1] Kalampokas E., Kalampokas T., Damaskos C. (2017). Primary Vaginal Melanoma, a Rare and Aggressive Entity. A Case Report and Review of the Literature. In Vivo.

[2] de Vries E., Bray F.I., Coebergh J.W.W., Parkin D.M. (2003). Changing Epidemiology of Malignant Cutaneous Melanoma in Europe 1953–1997: Rising Trends in Incidence and Mortality but Recent Stabilizations in Western Europe and Decreases in Scandinavia. Int. J. Cancer.

[3] Stang A., Pukkala E., Sankila R., Söderman B., Hakulinen T. (2006). Time Trend Analysis of the Skin Melanoma Incidence of Finland From 1953 Through 2003 Including 16,414 Cases. Int. J. Cancer.

[4] Koh D., Wang H., Lee J., Chia K.S., Lee H.P., Goh C.L. (2003). Basal Cell Carcinoma, Squamous Cell Carcinoma and Melanoma of the Skin: Analysis of the Singapore Cancer Registry Data 1968–1997. Br. J. Dermatol..

[5] Domingues B., Lopes J.M., Soares P., Pópulo H. (2018). Melanoma Treatment in Review. Immuno Targets Ther..

[6] Chang J.W.C. (2013). Acral Melanoma: A Unique Disease in Asia. JAMA Dermatol..

[7] Borkowska A.M., Szumera-Ciećkiewicz A., Spałek M.J., Teterycz P., Czarnecka A.M., Rutkowski P.Ł. (2020). Clinicopathological Features and Prognostic Factors of Primary Acral Melanomas in Caucasians. J. Clin. Med..

[8] Guo J., Si L., Kong Y., Flaherty K.T., Xu X., Zhu Y., Corless C.L., Li L., Li H., Sheng X. (2011). Phase II, Open-Label, Single-Arm Trial of Imatinib Mesylate in Patients With Metastatic Melanoma Harboring c-Kit Mutation or Amplification. J. Clin. Oncol..

[9] Bai X., Mao L.L., Chi Z.H., Sheng X.N., Cui C.L., Kong Y., Dai J., Wang X., Li S.M., Tang B.X. (2017). BRAF Inhibitors: Efficacious and Tolerable in BRAF-Mutant Acral and Mucosal Melanoma. Neoplasma.

[10] Nakamura Y., Namikawa K., Yoshino K., Yoshikawa S., Uchi H., Goto K., Nakamura Y., Fukushima S., Kiniwa Y., Takenouchi T. (2020). Anti-PD1 Checkpoint Inhibitor Therapy in Acral Melanoma: A Multicenter Study of 193 Japanese Patients. Ann. Oncol..

[11] Hall K.H., Rapini R.P. (2021). Acral Lentiginous Melanoma. StatPearls.

[12] Bristow I.R., Acland K. (2008). Acral Lentiginous Melanoma of the Foot and Ankle: A Case Series and Review of the Literature. J. Foot Ankle Res..

[13] Albreski D., Sloan S.B. (2009). Melanoma of the Feet: Misdiagnosed and Misunderstood. Clin. Dermatol..

[14] Bristow I.R., de Berker D.A., Acland K.M., Turner R.J., Bowling J. (2010). Clinical Guidelines for the Recognition of Melanoma of the Foot and Nail Unit. J. Foot Ankle Res..

[15] Stalkup J.R., Orengo I.F., Katta R. (2002). Controversies in Acral Lentiginous Melanoma. Dermatol. Surg..

[16] Kwon I.H., Lee J.H., Cho K.H. (2004). Acral Lentiginous Melanoma In Situ: A Study of Nine Cases. Am. J. Dermatopathol..

[17] Borkowska A., Szumera-Ciećkiewicz A., Spałek M., Teterycz P., Czarnecka A., Kowalik A., Rutkowski P. (2020). Mutation profile of primary subungual melanomas in Caucasians. Oncotarget.

[18] Gandini S., Sera F., Cattaruzza M.S., Pasquini P., Picconi O., Boyle P., Melchi C.F. (2005). Meta-Analysis of Risk Factors for Cutaneous Melanoma: II. Sun Exposure. Eur. J. Cancer.

[19] Hodis E., Watson I.R., Kryukov G.V., Arold S.T., Imielinski M., Theurillat J.P., Nickerson E., Auclair D., Li L., Place C. (2012). A Landscape of Driver Mutations in Melanoma. Cell.

[20] Chang Y.M., Barrett J.H., Bishop D.T., Armstrong B.K., Bataille V., Bergman W., Berwick M., Bracci P.M., Elwood J.M., Ernstoff M.S. (2009). Sun Exposure and Melanoma Risk at Different Latitudes: A Pooled Analysis of 5700 Cases and 7216 Controls. Int. J. Epidemiol..

[21] Curtin J.A., Fridlyand J., Kageshita T., Patel H.N., Busam K.J., Kutzner H., Cho K.H., Aiba S., Bröcker E.B., LeBoit P.E. (2005). Distinct Sets of Genetic Alterations in Melanoma. N. Engl. J. Med..

[22] Wittekind C., Neid M. (2005). Cancer Invasion and Metastasis. Oncology.

[23] Krakhmal N.V., Zavyalova M.V., Denisov E.V., Vtorushin S.V., Perelmuter V.M. (2015). Cancer Invasion: Patterns and Mechanisms. Acta Nat..

[24] Geiger T.R., Peeper D.S. (2009). Metastasis Mechanisms. Biochim. Biophys. Acta.

[25] Cheng G., Tse J., Jain R.K., Munn L.L. (2009). Micro-Environmental Mechanical Stress Controls Tumor Spheroid Size and Morphology by Suppressing Proliferation and Inducing Apoptosis in Cancer Cells. PLoS ONE.

[26] Tse J.M., Cheng G., Tyrrell J.A., Wilcox-Adelman S.A., Boucher Y., Jain R.K., Munn L.L. (2012). Mechanical Compression Drives Cancer Cells Toward Invasive Phenotype. Proc. Natl. Acad. Sci. USA.

[27] Stucke S., McFarland D., Goss L., Fonov S., McMillan G.R., Tucker A., Berme N., Cenk Guler H., Bigelow C., Davis B.L. (2012). Spatial Relationships Between Shearing Stresses and Pressure on the Plantar Skin Surface During Gait. J. Biomech..

[28] Minagawa A., Omodaka T., Okuyama R. (2016). Melanomas and Mechanical Stress Points on the Plantar Surface of the Foot. N. Engl. J. Med..

[29] Morikura T., Miyata S. (2019). Effect of Mechanical Compression on Invasion Process of Malignant Melanoma Using In Vitro Three-Dimensional Cell Culture Device. Micromachines.

[30] Chu T.C., Ranson W.F., Sutton M.A. (1985). Applications of Digital-Image-Correlation Techniques to Experimental Mechanics. Exp. Mech..

[31] Sutton M., Mingqi C., Peters W., Chao Y., McNeill S. (1986). Application of an Optimized Digital Correlation Method to Planar Deformation Analysis. Image Vis. Comput..

[32] Blaber J., Adair B., Antoniou A. (2015). Ncorr: Open-Source 2D Digital Image Correlation MATLAB Software. Exp. Mech..

[33] Ortiz J.S.E., Lagos R.E. (2015). A Viscoelastic Model to Simulate Soft Tissue Materials. J. Phys. Conf. Ser..

[34] Jayabal H., Dingari N.N., Rai B. (2019). A Linear Viscoelastic Model to Understand Skin Mechanical Behaviour and for Cosmetic Formulation Design. Int. J. Cosmet. Sci..

[35] Thakur V., Bedogni B. (2016). The Membrane Tethered Matrix Metalloproteinase MT1-MMP at the Forefront of Melanoma Cell Invasion and Metastasis. Pharmacol. Res..

[36] Barber R.D., Harmer D.W., Coleman R.A., Clark B.J. (2005). GAPDH as a Housekeeping Gene: Analysis of GAPDH mRNA Expression in a Panel of 72 Human Tissues. Physiol. Genom..

[37] Pollard T.D., Borisy G.G. (2003). Cellular Motility Driven by Assembly and Disassembly of Actin Filaments. Cell.

[38] Carlier M.F., Pantaloni D. (2007). Control of Actin Assembly Dynamics in Cell Motility. J. Biol. Chem..

[39] Jacquemet G., Hamidi H., Ivaska J. (2015). Filopodia in Cell Adhesion, 3D Migration and Cancer Cell Invasion. Curr. Opin. Cell Biol..

[40] Woodrum D.T., Rich S.A., Pollard T.D. (1975). Evidence for Biased Bidirectional Polymerization of Actin Filaments Using Heavy Meromyosin Prepared by an Improved Method. J. Cell Biol..

[41] Pollard T.D., Cooper J.A. (1986). Actin and Actin-Binding Proteins. A Critical Evaluation of Mechanisms and Functions. Annu. Rev. Biochem..

[42] Suetsugu S. (2014). Shaping the Membrane at Submicron Scale by BAR Proteins and the Actin Cytoskeleton. Seikagaku.

[43] Gardel M.L., Schneider I.C., Aratyn-Schaus Y., Waterman C.M. (2010). Mechanical Integration of Actin and Adhesion Dynamics in Cell Migration. Annu. Rev. Cell Dev. Biol..

[44] Peng H. (2008). Bioimage informatics: A new area of engineering biology. Bioinformatics.

[45] Altadill A., Rodríguez M., González L.O., Junquera S., Corte M.D., González-Dieguez M.L., Linares A., Barbón E., Fresno-Forcelledo M., Rodrigo L. (2009). Liver expression of matrix metalloproteases and their inhibitors in hepatocellular carcinoma. Dig. Liver Dis..

[46] Hotary K.B., Allen E.D., Brooks P.C., Datta N.S., Long M.W., Weiss S.J. (2003). Membrane type I matrix metalloproteinase usurps tumor growth control imposed by the three-dimensional extracellular matrix. Cell.

[47] Koshikawa N., Minegishi T., Sharabi A., Quaranta V., Seiki M. (2005). Membrane-type matrix metalloproteinase-1 (MT1-MMP) is a processing enzyme for human laminin gamma 2 chain. J. Biol. Chem..

[48] Seiki M. (2002). The cell surface: The stage for matrix metalloproteinase regulation of migration. Curr. Opin. Cell Biol..

[49] Shaverdashvili K., Wong P., Ma J., Zhang K., Osman I., Bedogni B. (2014). MT1-MMP modulates melanoma cell dissemination and metastasis through activation of MMP2 and RAC1. Pigment. Cell Melanoma Res..

[50] Sato H., Takino T., Okada Y., Cao J., Shinagawa A., Yamamoto E., Seiki M. (1994). A matrix metalloproteinase expressed on the surface of invasive tumour cells. Nature.

[51] Ranjan A., Kalraiya R.D. (2014). Invasive potential of melanoma cells correlates with the expression of MT1-MMP and regulated by modulating its association with motility receptors via N-glycosylation on the receptors. Biomed. Res. Int..

